# Associations of Vegetable and Potato Intakes With Markers of Type 2 Diabetes Risk in the AusDiab Cohort

**DOI:** 10.1210/clinem/dgae333

**Published:** 2024-05-15

**Authors:** Pratik Pokharel, Lauren C Blekkenhorst, Catherine P Bondonno, Kevin Murray, Simone Radavelli-Bagatini, Dianna J Magliano, Robin M Daly, Jonathan E Shaw, Joshua R Lewis, Jonathan M Hodgson, Nicola P Bondonno

**Affiliations:** Nutrition and Health Innovation Research Institute, School of Medical and Health Sciences, Edith Cowan University, Perth, Western Australia 6000, Australia; Diet Cancer and Health Group, Danish Cancer Institute, Copenhagen 2100, Denmark; Nutrition and Health Innovation Research Institute, School of Medical and Health Sciences, Edith Cowan University, Perth, Western Australia 6000, Australia; Medical School, University of Western Australia, Royal Perth Hospital, Perth, Western Australia 6000, Australia; Nutrition and Health Innovation Research Institute, School of Medical and Health Sciences, Edith Cowan University, Perth, Western Australia 6000, Australia; Medical School, University of Western Australia, Royal Perth Hospital, Perth, Western Australia 6000, Australia; School of Population and Global Health, University of Western Australia, Perth, Western Australia 6009, Australia; Nutrition and Health Innovation Research Institute, School of Medical and Health Sciences, Edith Cowan University, Perth, Western Australia 6000, Australia; Department of Diabetes and Population Health, Baker Heart and Diabetes Institute (HDI), Melbourne, Victoria 3004, Australia; Institute for Physical Activity and Nutrition, School of Exercise and Nutrition Sciences, Deakin University, Geelong, Victoria 3220, Australia; Department of Diabetes and Population Health, Baker Heart and Diabetes Institute (HDI), Melbourne, Victoria 3004, Australia; Department of Epidemiology and Preventive Medicine, Monash University, Melbourne, Victoria 3170, Australia; Nutrition and Health Innovation Research Institute, School of Medical and Health Sciences, Edith Cowan University, Perth, Western Australia 6000, Australia; Medical School, University of Western Australia, Royal Perth Hospital, Perth, Western Australia 6000, Australia; Nutrition and Health Innovation Research Institute, School of Medical and Health Sciences, Edith Cowan University, Perth, Western Australia 6000, Australia; Medical School, University of Western Australia, Royal Perth Hospital, Perth, Western Australia 6000, Australia; Nutrition and Health Innovation Research Institute, School of Medical and Health Sciences, Edith Cowan University, Perth, Western Australia 6000, Australia; Diet Cancer and Health Group, Danish Cancer Institute, Copenhagen 2100, Denmark; School of Biomedical Sciences, University of Western Australia, Royal Perth Hospital, Perth, Western Australia 6000, Australia

**Keywords:** leafy vegetable, green vegetables, Brassica vegetables, nutrition epidemiology, type 2 diabetes mellitus, diabetes markers

## Abstract

**Context:**

The associations of vegetable and potato intakes with type 2 diabetes (T2D) appear to be nuanced, depending on vegetable types and preparation method, respectively.

**Objective:**

We investigated the associations of total vegetable, vegetable subgroup, and potato intakes with (1) markers of T2D at baseline and (2) incident T2D cumulative over a 12-year follow-up period in Australian adults.

**Methods:**

Using data from the Australian Diabetes, Obesity and Lifestyle Study, intakes of vegetables and potatoes were assessed via a food frequency questionnaire at baseline. Associations between vegetable intake and (1) fasting plasma glucose (FPG), 2-hour postload plasma glucose (PLG), updated homeostasis model assessment of β-cell function (HOMA2-%β), HOMA2 of insulin sensitivity (HOMA2-%S), and fasting insulin levels at baseline; and (2) cumulative incident T2D at the end of 12-year follow-up were examined using generalized linear and Cox proportional hazards models, respectively.

**Results:**

In total, 8009 participants were included having median age of 52 years, and vegetable intake of 132 g/day. Higher intake of total vegetable, green leafy, yellow/orange/red, and moderate intakes of cruciferous vegetables was associated with lower PLG. Additionally, higher green leafy vegetable intake was associated with lower HOMA2-%β and serum insulin. Conversely, higher potato fries/chips intakes were associated with higher FPG, HOMA2-%β, serum insulin, and lower HOMA2-%S. Participants with moderate cruciferous vegetables intake had a 25% lower risk of T2D at the end of 12 years of follow-up.

**Conclusion:**

A higher intake of vegetables, particularly green leafy vegetables, may improve while consuming potato fries/chips, but not potatoes prepared in a healthy way, may worsen glucose tolerance and insulin sensitivity. Our findings suggest a nuanced relationship between vegetable subgroups and their impact on glucose tolerance.

The global burden of type 2 diabetes (T2D) increased to about 507 million in 2021, underscoring the persistent challenge for its prevention (1). T2D is strongly associated with several modifiable risk factors including poor diet, which, alone, accounted for 14.1 million new cases in 2018 (2). While intakes of ultraprocessed foods, red and processed meat, and refined grains are linked to a higher risk of T2D (3-5), healthy plant-based foods are recommended to mitigate the risk of T2D (6). Our previous research, alongside other studies, underscores the importance of vegetables, in particular green leafy and cruciferous vegetables, in lowering the risk of T2D (4, 7-9). Less clear is the role that potatoes—a dietary staple which are considered to be a vegetable in the current Australian Dietary Guidelines (10)—play in the development of T2D. Potatoes have been linked to a higher risk of T2D (4, 11), but these positive associations are likely influenced by preparation methods and underlying dietary patterns (9). While potatoes are recommended as part of an environmentally sustainable and healthy diet (12, 13), our research indicates that substituting 25 g/day of potatoes with an equivalent amount of green leafy or cruciferous vegetables is associated with 21% and 13% lower risk of T2D, respectively (manuscript submitted for publication), highlighting potential distinctions in the metabolic impact between specific vegetable subtypes and potatoes.

In Australia, data from the 2022 national survey showed that only 6.5% of the adults met the recommended daily intake of at least 5 servings of vegetables, reflecting a decline from 7.5% in 2017-2018 (14). Concurrently, the prevalence of diabetes in Australia has risen from 3.3% in 2001 to 5.3% in 2022 (15). Obesity has emerged as the foremost risk factor among the 16 recognized risk factors for T2D worldwide (1); two-thirds (65.8%) of Australian adults were classified as overweight or obese in 2022 (16). Promoting increased vegetable consumption would be a valuable strategy to reduce the burden of T2D in Australia. However, given the ongoing challenge of low vegetable intake, an alternative approach might involve advocating for the prioritization of intake of specific vegetable subtypes known for their superior health benefits. While the role of glucose metabolism (reflected by markers of insulin resistance and β-cell dysfunction) in the development of T2D is well-established, the intricate relationship between vegetable intakes, including potatoes and these subclinical markers remains uncertain. Investigating these relationships may provide valuable insights into the mechanisms through which a higher intake of vegetables or vegetable subgroups may confer the greatest protection against T2D, while also shedding light on the nuanced impact of potatoes. Therefore, the primary aim of this population-based study was to examine the association of total vegetable, vegetable subgroups, and potato intakes with measures of insulin resistance and β-cell dysfunction. The secondary aim was to examine the association of these exposures with incident T2D at 12 years of follow-up in Australian adults.

## Materials and Methods

### Study Participants

The study included individuals from the Australian Diabetes, Obesity and Lifestyle (AusDiab) study, a national population-based cohort study of the prevalence and impact of diabetes in Australia. Details regarding the methodology of this cohort are available elsewhere (17). In brief, the AusDiab study invited 17 129 eligible participants aged ≥ 25 years across all the states and the Northern Territory of Australia, culminating in a cohort of 11 247 adults (5049 men and 6198 women) who underwent a complete biomedical examination during 1999-2001. Subsequently, these participants were followed up at wave 1 in 2004-2005 (n = 6400) and wave 2 in 2011-2012 (n = 4614) (18). From the original baseline cohort of 11 247 participants, we excluded those with known diabetes prior to the baseline assessment (n = 578), pregnant women (n = 45), those with implausible energy intake (n = 342) at baseline (<3300 kJ/day or >17 500 kJ/day for men and <2500 kJ/day or >14 500 kJ/day for women) (19, 20), as well as those with missing data on exposures (n = 204), covariates (n = 698) and outcomes (n = 1371); the final cohort for the current study was 8009 participants for initial analyses (Fig. S1 (21)). Of these, 5107 participants had data on diabetes status at either wave 1 and/or wave 2. All participants provided written informed consent. Approval for the study was obtained from the Human Research Ethics Committees of the International Diabetes Institute and the Alfred Hospital, Melbourne, Australia.

### Exposure Assessment

In order to assess participants' usual eating habits over the 12 months prior to baseline, a self-administered 74-item Food Frequency Questionnaire (FFQ) developed by the Cancer Council Victoria was used (22, 23). For the FFQ, participants indicated their usual frequency of food item consumption, selecting from 10 frequency response options, ranging from “never” to “three or more times per day.” Recognizing the potential for overestimation in self-reported intakes, additional questions on total frequency of intake of foods were utilized to correct the results. The determination of portion sizes utilized photographs depicting scaled portions of various food types. This FFQ was compared with 7-day weighed food records in 63 women of childbearing age from a different study on iron supplementation, with energy-adjusted correlation coefficients of 0.72 for fiber, 0.60 for vitamin C and 0.64 for β-carotene (22).

The exposures of interest for this study were intakes of total vegetables, subgroups of vegetables, and potatoes reported in the FFQ at baseline. The classification of vegetable subgroups adhered to guidelines from both the American and Australian Dietary Guidelines (10, 24). These subgroups included green leafy vegetables (lettuce, endive, other salad greens, silverbeet, spinach, celery); cruciferous vegetables (cabbage, Brussels sprouts, cauliflower, broccoli); allium vegetables (onion, leek, and garlic); yellow/orange/red vegetables (tomatoes, carrots, pumpkin, capsicum, beetroot, tomato sauce, tomato paste, dried tomatoes); legumes (green beans, bean sprouts, alfalfa sprouts, baked beans, soy beans, soy bean curd, tofu, peas, and other beans) and other vegetables (cucumber, mushroom, avocado, zucchini). Total vegetable intakes were calculated as the sum of the intakes of each vegetable described above. For intakes of potatoes, we differentiated between total potatoes (excluding potato fries/chips) and the specific category of potato fries/chips.

### Outcome Assessment

Following an overnight fast lasting ≥ 8 hours, participants provided fasting blood samples and underwent a standardized 75-g oral glucose tolerance test (25). The resultant biochemical tests constituted the basis for the primary outcomes in this study incorporating metabolic markers. These markers included fasting plasma glucose (FPG), 2-hour postload plasma glucose (PLG), updated homeostasis model assessment of β-cell function (HOMA2-%β), HOMA2 of insulin sensitivity (HOMA2-%S), and fasting insulin levels, all measured at baseline. FPG and PLG were determined with a glucose oxidase method (18), while serum insulin was quantified using an automated chemiluminescence immunoassay. The HOMA2 computer model was used to estimate insulin sensitivity (HOMA2-%S) and β-cell function (HOMA2-%β) from fasting insulin and glucose concentrations (26). As a secondary outcome, the study investigated the association of vegetable and potato intakes with T2D (cumulative incidence) at the end of 12 years of follow-up. Incident T2D was defined as FPG level of ≥7.0 mmol/L (≥126 mg/dL), 2-hour PLG level of ≥11.1 mmol/L (≥200 mg/dL), or treatment with insulin or oral hypoglycemic agents (27).

### Assessment of Covariates

Data on demographic and health-related information were collected from interviewer-administered questionnaires (18). Participants provided details on key demographic factors, including sex (male/female), education level (never to some high school, completed university or equivalent), physical activity level (categorized as sedentary = 0 minutes/week, insufficient = 1-149 minutes/week, and sufficient ≥150 minutes/week) based on the Active Australia Survey (28), smoking status (nonsmoker, ex-smoker, current smoker), income, parental history of diabetes (yes/no), and self-reported prevalence of cardiovascular disease (yes/no). Weight was assessed without shoes and excess clothing using a mechanical beam balance (17, 29). Body mass index (BMI) was calculated as the ratio of weight (in kilograms) to height (in meters) squared. The Socio-Economic Indexes for Areas (SEIFA) information was obtained as reported by the Australian Bureau of Statistics (30). Additionally, energy intake (kcal/day) and intakes of other dietary covariates, including wholegrains, refined grains and more were estimated from the 75 food items included in the FFQ (23).

### Statistical Analysis

The baseline characteristics of participants were summarized, both overall and stratified by quartiles of total vegetable intake. Two types of analyses were conducted: a cross-sectional analysis and a time to event analysis. To explore cross-sectional associations with the continuous exposures at baseline, a generalized linear model with a gamma distribution and log-link function was employed, given the non-negative and continuous nature of the primary outcomes. To assess potential nonlinear associations, exposures were modeled using restricted cubic splines. The “rms” R package facilitated this analysis, where the exposure was fitted as a continuous variable through a restricted cubic spline and are reported for the median intake in each quartile (Q2-Q4) relative to the median intake in the lowest quartile (Q1) to demonstrate comparison between quartiles of intake. For the cross-sectional analyses, the β coefficients in the tables are the differences in log means of the higher quartile with the reference but are exponentiated to present as a ratio of means in the results for interpretation. The graphical representation of these cross-sectional associations for the dose–response relationship was achieved using the “effects” R package. For the time to event analysis, Cox proportional hazards models were employed to investigate associations between baseline exposure intake and incident T2D over a 12-year follow-up period. Age of the participants served as the underlying timescale were as follows: for participants without any record of T2D during follow-up, age at the latest follow-up was used for follow-up time; for participants with a record of T2D at wave 1, age at the midpoint between study entry and wave 1 was used; for participants with a record of T2D at wave 2 only, age at the midpoint between wave 1 and wave 2 was used. We did not have any participants who missed wave 1 and attended wave 2. For this time to event analysis, the continuous exposures were modeled as restricted cubic splines, with the median intake of the first quartile used as the reference for comparisons to describe potential nonlinear relationships with incident T2D. In graphs depicting associations, HRs and 95% CIs were graphed on the y-axis, with exposures on the x-axis and median intake in Q1 set as the reference. For better data illustration, all exposures were restricted at 3 SD above the mean. Assumptions of the Cox model was confirmed by visually inspecting the parallel appearance of the log-log plots of the survival function, revealing no violations. In both the cross-sectional and time to event analyses, *P* values for the effect of exposures on each of the responses and for a test of nonlinearity were obtained using likelihood ratio tests comparing appropriate nested models. Confounding factors were selected a priori based on their relationship with the outcome and exposure. The following models were predetermined for adjustments: Model 1a adjusted for age (in years) and sex; Model 1b adjusted for age, sex, education level, physical activity level, smoking status, SEIFA score, income, parental history of diabetes, and alcohol intake (g/day); Model 2 adjusted for all covariates in Model 1b plus intakes (g/day) of wholegrains, refined grains, red meat, processed meat, poultry, eggs, fish, dairy, fat, fruits, nuts, and miscellaneous and discretionary foods (all-component model approach (31)); Model 3 adjusted for all covariates in Model 2 plus BMI (kg/m^2^) and self-reported prevalence of hypertension (yes/no), high cholesterol (yes/no), and cardiovascular disease (yes/no). To investigate whether the cross-sectional associations were modified by sex (male/female), BMI (<30 and ≥30), and physical activity level (sedentary, insufficient, and sufficient), multiplicative interaction terms were added to Model 1b separately, and interaction *P* values were obtained using likelihood ratio tests comparing nested models. The statistical analyses were conducted using R statistics (R Core Team, 2023 (32)) with all *P* values being 2 tailed, and the statistical significance level set at 0.05.

## Results

### Participant Characteristics

For the 8009 participants in this analytic cohort, the median (interquartile range, IQR) age was 52 (44-63) years, and 45% were male (Table 1). The median vegetable intake for this cohort was 132 (98-174) g/day. A higher proportion of participants in the highest (Q4) quartile of vegetable intake compared with those in the lowest quartile (Q1) were female, marginally more disadvantaged, possessed a higher education, engaged more in physical activity, and were less likely to be smokers. Furthermore, those in the highest vegetable intake category tended to have a higher daily energy intake, primarily driven by a higher consumption across all other food categories. Upon energy adjustment, participants in the highest compared with the lowest vegetable intake category had higher intake of wholegrains, potatoes, fruits, red meat, poultry, fish, and nuts, along with lower intakes of refined grains, eggs, and discretionary foods (Fig. S2 (21)). The vegetable-eating habit of this population is characterized by a higher preference for yellow/orange/red vegetables (39 [26-55] g/day), followed by legumes (26 [16-40] g/day), cruciferous vegetables (21 [11-34] g/day), other vegetables (17 [9-28] g/day), green leafy vegetables (12 [7-19] g/day), and allium vegetables (5 [2-9] g/day). Participants with no follow-up data after baseline tended to have a slightly lower intake of vegetables, were marginally disadvantaged, were more likely to be current smokers, possessed a lower educational degree, and had a lower intake of most of the foods and energy intake than those with follow-up (Table S1 (21)).

**Table 1.** Baseline characteristics of the study population by quartiles of vegetable intake

	Total population (n = 8009)	Total vegetable intake quartiles*^a^*			
		Quartile 1	Quartile 2	Quartile 3	Quartile 4
		(n = 2006)	(n = 1999)	(n = 2002)	(n = 2002)
Total vegetable intake, g/d	132 (98, 174)	75 (57, 88)	114 (106, 123)	150 (141, 161)	208 (189, 242)
Sex, male, n (%)	3607 (45.0)	896 (44.7)	824 (41.2)	938 (46.9)	949 (47.4)
Age, years	52 (44, 63)	52 (44, 64)	52 (44, 63)	52 (45, 63)	53 (45, 62)
BMI, kg/m^2^	26.4 (23.7, 29.5)	26.1 (23.4, 29.2)	26.4 (23.6, 29.5)	26.5 (23.9, 29.5)	26.4 (23.8, 29.6)
SEIFA score	1033 (967, 1079)	1033 (974, 1079)	1044 (977, 1081)	1032 (967, 1075)	1027 (964, 1075)
Physical activity, n (%)					
Sedentary (0 minutes/week)	1387 (17.3)	423 (21.1)	337 (16.9)	328 (16.4)	299 (14.9)
Insufficient (<150 minutes/week)	2497 (31.2)	667 (33.3)	639 (32.0)	635 (31.7)	556 (27.8)
Sufficient (≥150 minutes/week)	4125 (51.5)	916 (45.7)	1023 (51.2)	1039 (51.9)	1147 (57.3)
Smoking status, n (%)					
Current	1135 (14.2)	321 (16.0)	273 (13.7)	277 (13.8)	264 (13.2)
Former	2429 (30.3)	573 (28.6)	574 (28.7)	627 (31.3)	655 (32.7)
Never	4445 (55.5)	1112 (55.4)	1152 (57.6)	1098 (54.8)	1083 (54.1)
Education, n (%)					
Never, primary, or high school	3281 (41.0)	827 (41.2)	833 (41.7)	850 (42.5)	771 (38.5)
University or equivalent	4728 (59.0)	1179 (58.8)	1166 (58.3)	1152 (57.5)	1231 (61.5)
Prevalent CVD, n (%)	671 (8.4)	161 (8.0)	162 (8.1)	158 (7.9)	190 (9.5)
Family history of diabetes, n (%)	1459 (18.2)	378 (18.8)	354 (17.7)	369 (18.4)	358 (17.9)
Prevalent hypertension, n (%)	2729 (34.1)	666 (33.2)	683 (34.2)	693 (34.6)	687 (34.3)
Prevalent high cholesterol, n (%)	5127 (64.0)	1303 (65.0)	1333 (66.7)	1254 (62.6)	1237 (61.8)
Dietary characteristics					
Energy, kcal/d	1872 (1471, 2352)	1633 (1277, 2077)	1759 (1394, 2147)	1974 (1586, 2431)	2159 (1726, 2702)
Alcohol, g/d	6 (1, 19)	4 (1, 15)	6 (1, 18)	7 (1, 21)	6 (1, 20)
Wholegrains, g/d	83 (26, 147)	57 (9, 120)	76 (27, 134)	91 (31, 155)	108 (52, 185)
Refined grains, g/d	124 (72, 190)	119 (67, 186)	114 (68, 175)	129 (72, 195)	135 (82, 205)
Red meat, g/d	59 (34, 96)	47 (26, 79)	55 (32, 83)	66 (40, 105)	75 (44, 120)
Processed meat, g/d	17 (8, 31)	14 (6, 28)	16 (8, 28)	19 (9, 33)	18 (7, 34)
Poultry, g/d	22 (13, 38)	19 (10, 33)	22 (12, 34)	26 (14, 42)	29 (16, 46)
Dairy, g/d	328 (211, 414)	277 (208, 404)	328 (214, 410)	354 (214, 418)	354 (212, 426)
Fish, g/d	26 (14, 44)	20 (10, 35)	23 (13, 38)	28 (16, 46)	34 (19, 57)
Potato, g/d	32 (13, 57)	15 (5, 32)	29 (14, 49)	39 (19, 61)	52 (27, 81)
Fruit, g/d	181 (101, 300)	129 (77, 239)	162 (102, 265)	200 (112, 313)	248 (128, 376)
Fats, g/d	21 (14, 28)	14 (7, 28)	21 (14, 28)	21 (14, 28)	21 (7, 28)

Values are presented as medians (interquartile range) or n (%), unless otherwise stated.

Abbreviations: BMI, body mass index; CVD, cardiovascular disease; SEIFA, Socio-Economic Indexes for Areas.

^*a*^Total vegetable intake excluding potatoes.

### Cross-sectional Association Between Vegetable Intake and Markers of T2D

**Figure 1.**
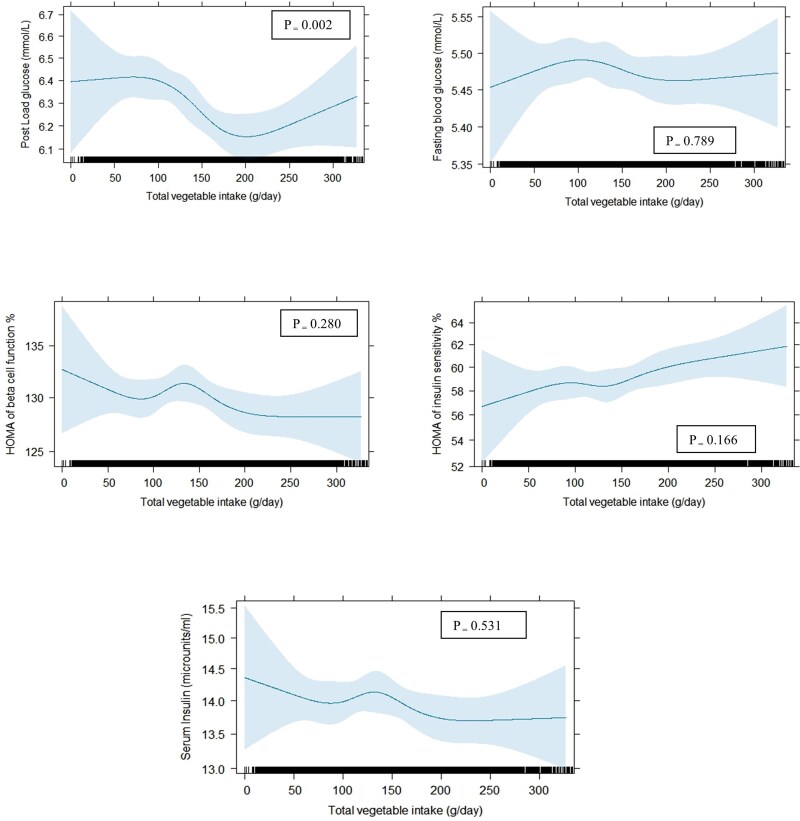
The graph based on restricted cubic splines for the association between total vegetable intake and markers of T2D at baseline: (A) 2-hour post load plasma glucose; (B) fasting blood glucose; (C) updated homeostasis model assessment (HOMA2) of β-cell function; (D) HOMA2 of insulin sensitivity; and (E) fasting serum insulin, obtained by generalized linear model (n = 8009). All the analyses were adjusted for age, sex, physical activity level, education level, Socio-Economic Indexes for Areas, income, smoking status, parental history of diabetes and alcohol intake. The blue shaded area represents 95% CI. *P* values for the overall effect of vegetable intakes on the response were obtained using likelihood ratio tests.

Total vegetable intake was significantly associated with PLG (*P* = .002; *P*_nonlinearity_ = .038; Model 1b; Table 2 and Fig. 1) upon multivariable adjustments but not with FPG, serum insulin, HOMA2-%β, and HOMA2-%S (Table 2; Table 3 and Fig. 1). However, this association with PLG was inverse only over a range of 100 to 200 g of vegetables per day. Participants in the highest quartile compared with the lowest vegetable intake had a 3% (ratio of means 0.97 [95% CI 0.96, 0.99]) lower PLG upon multivariable adjustments (Model 1b; Table 2). This association remained significant upon further adjustment for dietary confounders (Model 2) as well as potential mediators (Model 3; Table 2).

**Table 2.** Association of vegetables intake with postload plasma glucose and fasting blood glucose (n = 8009)

	Quartiles of vegetable intake			
	Q1	Q2	Q3	Q4
**PLG, mmol/L**				
Total vegetables				
Intake, g/day	75 (57-88)	114 (106-123)	150 (141-161)	208 (189-242)
Model 1a	Reference	−0.016 (−0.026, −0.005)	−0.025 (−0.040, −0.010)	−0.031 (−0.048, −0.014)
Model 1b	Reference	−0.014 (−0.024, −0.003)	−0.022 (−0.037, −0.007)	−0.027 (−0.044, −0.010)
Model 2	Reference	−0.011 (−0.022, −0.001)	−0.018 (−0.033, −0.003)	−0.023 (−0.040, −0.005)
Model 3	Reference	−0.014 (−0.023, −0.004)	−0.022 (−0.036, −0.007)	−0.025 (−0.042, −0.008)
Cruciferous vegetables				
Intake, g/day	6 (3-8)	16 (13-18)	26 (23-30)	47 (39-58)
Model 1a	Reference	−0.014 (−0.024, −0.003)	−0.022 (−0.040, −0.005)	−0.019 (−0.038, 0.000)
Model 1b	Reference	−0.012 (−0.022, −0.002)	−0.020 (−0.037, −0.003)	−0.018 (−0.036, 0.001)
Model 2	Reference	−0.011 (−0.021, −0.001)	−0.018 (−0.035, −0.001)	−0.015 (−0.034, 0.003)
Model 3	Reference	−0.011 (−0.021, −0.001)	−0.019 (−0.035, −0.002)	−0.017 (−0.035, 0.001)
Green leafy vegetables				
Intake, g/day	4 (2-6)	10 (8-11)	15 (14-17)	26 (22-32)
Model 1a	Reference	−0.023 (−0.034, −0.012)	−0.037 (−0.054, −0.020)	−0.044 (−0.062, −0.025)
Model 1b	Reference	−0.019 (−0.030, −0.008)	−0.031 (−0.048, −0.014)	−0.036 (−0.055, −0.018)
Model 2	Reference	−0.019 (−0.030, −0.008)	−0.031 (−0.049, −0.014)	−0.037 (−0.056, −0.018)
Model 3	Reference	−0.018 (−0.028, −0.007)	−0.029 (−0.045, −0.013)	−0.035 (−0.054, −0.017)
Legumes				
Intake, g/day	10 (6-13)	21 (18-23)	32 (29-36)	52 (45-65)
Model 1a	Reference	−0.009 (−0.019, 0.002)	−0.013 (−0.030, 0.004)	−0.008 (−0.026, 0.011)
Model 1b	Reference	−0.008 (−0.018, 0.002)	−0.012 (−0.029, 0.004)	−0.008 (−0.026, 0.010)
Model 2	Reference	−0.006 (−0.016, 0.005)	−0.008 (−0.024, 0.009)	−0.002 (−0.020, 0.016)
Model 3	Reference	−0.007 (−0.016, 0.003)	−0.010 (−0.026, 0.006)	−0.006 (−0.023, 0.012)
Yellow/orange/red vegetables				
Intake, g/day	17 (11-22)	32 (29-35)	46 (42-50)	70 (62-83)
Model 1a	Reference	−0.025 (−0.036, −0.014)	−0.039 (−0.055, −0.023)	−0.040 (−0.058, −0.023)
Model 1b	Reference	−0.023 (−0.034, −0.012)	−0.036 (−0.052, −0.020)	−0.038 (−0.056, −0.021)
Model 2	Reference	−0.019 (−0.030, −0.008)	−0.030 (−0.046, −0.013)	−0.030 (−0.049, −0.012)
Model 3	Reference	−0.019 (−0.030, −0.009)	−0.030 (−0.046, −0.014)	−0.029 (−0.047, −0.012)
Allium vegetables				
Intake, g/day	1 (0-2)	4 (3-4)	7 (6-8)	13 (11-17)
Model 1a	Reference	−0.011 (−0.021, −0.001)	−0.019 (−0.036, −0.002)	−0.015 (−0.034, 0.004)
Model 1b	Reference	−0.007 (−0.016, 0.003)	−0.011 (−0.028, 0.006)	−0.006 (−0.025, 0.013)
Model 2	Reference	−0.006 (−0.016, 0.003)	−0.011 (−0.028, 0.007)	−0.006 (−0.026, 0.013)
Model 3	Reference	−0.006 (−0.015, 0.003)	−0.010 (−0.027, 0.006)	−0.007 (−0.025, 0.012)
Other vegetables				
Intake, g/day	5 (3-7)	13 (11-15)	22 (19-25)	40 (33-51)
Model 1a	Reference	−0.023 (−0.033, −0.013)	−0.041 (−0.058, −0.024)	−0.052 (−0.071, −0.033)
Model 1b	Reference	−0.018 (−0.028, −0.008)	−0.032 (−0.049, −0.015)	−0.040 (−0.059, −0.021)
Model 2	Reference	−0.018 (−0.028, −0.007)	−0.031 (−0.048, −0.014)	−0.040 (−0.059, −0.020)
Model 3	Reference	−0.021 (−0.031, −0.011)	−0.037 (−0.053, −0.020)	−0.045 (−0.064, −0.026)
Potatoes				
Intake, g/day	5 (1-9)	22 (18-27)	43 (37-50)	81 (67-104)
Model 1a	Reference	−0.014 (−0.025, −0.004)	−0.024 (−0.042, −0.006)	−0.024 (−0.043, −0.004)
Model 1b	Reference	−0.013 (−0.023, −0.003)	−0.022 (−0.040, −0.005)	−0.023 (−0.042, −0.004)
Model 2	Reference	−0.009 (−0.019, 0.001)	−0.015 (−0.033, 0.003)	−0.013 (−0.033, 0.006)
Model 3	Reference	−0.009 (−0.019, 0.001)	−0.016 (−0.033, 0.002)	−0.014 (−0.033, 0.005)
Potato fries/chips				
Intake, g/day	2 (1-4)	8 (7-10)	17 (14-21)	42 (32-60)
Model 1a	Reference	0.002 (−0.005, 0.010)	0.006 (−0.010, 0.022)	0.018 (−0.004, 0.039)
Model 1b	Reference	0.001 (−0.006, 0.009)	0.004 (−0.012, 0.020)	0.014 (−0.007, 0.035)
Model 2	Reference	0.003 (−0.005, 0.011)	0.008 (−0.009, 0.025)	0.020 (−0.002, 0.043)
Model 3	Reference	0.001 (−0.007, 0.009)	0.003 (−0.013, 0.019)	0.012 (−0.009, 0.034)
**FPG, mmol/L**				
Total vegetables				
Intake, g/day	75 (57-88)	114 (106-123)	150 (141-161)	208 (189-242)
Model 1a	Reference	−0.001 (−0.005, 0.003)	−0.001 (−0.007, 0.004)	−0.003 (−0.009, 0.004)
Model 1b	Reference	0.000 (−0.004, 0.004)	−0.001 (−0.006, 0.005)	−0.002 (−0.008, 0.004)
Model 2	Reference	0.000 (−0.004, 0.004)	−0.001 (−0.006, 0.005)	−0.002 (−0.009, 0.005)
Model 3	Reference	−0.001 (−0.005, 0.003)	−0.002 (−0.007, 0.004)	−0.003 (−0.010, 0.003)
Cruciferous vegetables				
Intake, g/day	6 (3-8)	16 (13-18)	26 (23-30)	47 (39-58)
Model 1a	Reference	−0.002 (−0.006, 0.002)	−0.003 (−0.010, 0.003)	−0.001 (−0.008, 0.006)
Model 1b	Reference	−0.002 (−0.006, 0.002)	−0.002 (−0.009, 0.004)	0.000 (−0.007, 0.007)
Model 2	Reference	−0.002 (−0.006, 0.002)	−0.003 (−0.009, 0.004)	−0.001 (−0.008, 0.006)
Model 3	Reference	−0.011 (−0.021, −0.001)	−0.003 (−0.009, 0.003)	−0.001 (−0.008, 0.005)
Green leafy vegetables				
Intake, g/day	4 (2-6)	10 (8-11)	15 (14-17)	26 (22-32)
Model 1a	Reference	−0.003 (−0.007, 0.001)	−0.004 (−0.011, 0.002)	−0.004 (−0.011, 0.003)
Model 1b	Reference	−0.002 (−0.006, 0.002)	−0.003 (−0.009, 0.003)	−0.002 (−0.009, 0.005)
Model 2	Reference	−0.002 (−0.007, 0.002)	−0.004 (−0.010, 0.003)	−0.003 (−0.010, 0.004)
Model 3	Reference	−0.002 (−0.006, 0.002)	−0.003 (−0.009, 0.003)	−0.002 (−0.009, 0.005)
Legumes				
Intake, g/day	10 (6-13)	21 (18-23)	32 (29-36)	52 (45-65)
Model 1a	Reference	0.001 (−0.003, 0.005)	0.002 (−0.005, 0.008)	0.004 (−0.003, 0.010)
Model 1b	Reference	0.001 (−0.003, 0.005)	0.002 (−0.005, 0.008)	0.003 (−0.004, 0.010)
Model 2	Reference	0.001 (−0.003, 0.005)	0.002 (−0.004, 0.009)	0.004 (−0.003, 0.011)
Model 3	Reference	0.001 (−0.003, 0.005)	0.002 (−0.005, 0.008)	0.002 (−0.004, 0.009)
Yellow/orange/red vegetables				
Intake, g/day	17 (11-22)	32 (29-35)	46 (42-50)	70 (62-83)
Model 1a	Reference	−0.005 (−0.009, −0.001)	−0.008 (−0.014, −0.002)	−0.009 (−0.015, −0.002)
Model 1b	Reference	−0.005 (−0.009, 0.000)	−0.007 (−0.013, −0.001)	−0.008 (−0.015, −0.001)
Model 2	Reference	−0.004 (−0.008, 0.000)	−0.007 (−0.013, 0.000)	−0.007 (−0.014, 0.000)
Model 3	Reference	−0.004 (−0.008, 0.000)	−0.006 (−0.012, 0.000)	−0.007 (−0.013, 0.000)
Allium vegetables				
Intake, g/day	1 (0-2)	4 (3-4)	7 (6-8)	13 (11-17)
Model 1a	Reference	0 (−0.003, 0.004)	0.001 (−0.006, 0.007)	0.001 (−0.006, 0.008)
Model 1b	Reference	0.001 (−0.003, 0.005)	0.002 (−0.005, 0.008)	0.002 (−0.005, 0.009)
Model 2	Reference	0.001 (−0.003, 0.005)	0.002 (−0.005, 0.008)	0.001 (−0.006, 0.009)
Model 3	Reference	0.001 (−0.003, 0.004)	0.002 (−0.005, 0.008)	0.001 (−0.006, 0.008)
Other vegetables				
Intake, g/day	5 (3-7)	13 (11-15)	22 (19-25)	40 (33-51)
Model 1a	Reference	−0.002 (−0.005, 0.002)	−0.003 (−0.010, 0.003)	−0.007 (−0.014, 0.001)
Model 1b	Reference	0.000 (−0.004, 0.003)	−0.001 (−0.008, 0.005)	−0.004 (−0.011, 0.003)
Model 2	Reference	−0.001 (−0.005, 0.003)	−0.002 (−0.009, 0.004)	−0.006 (−0.013, 0.002)
Model 3	Reference	−0.002 (−0.006, 0.002)	−0.004 (−0.010, 0.002)	−0.007 (−0.015, 0.000)
Potatoes				
Intake, g/day	5 (1-9)	22 (18-27)	43 (37-50)	81 (67-104)
Model 1a	Reference	−0.003 (−0.007, 0.001)	−0.005 (−0.011, 0.002)	−0.003 (−0.010, 0.004)
Model 1b	Reference	−0.003 (−0.007, 0.001)	−0.004 (−0.011, 0.002)	−0.003 (−0.010, 0.004)
Model 2	Reference	−0.002 (−0.006, 0.002)	−0.003 (−0.010, 0.004)	−0.001 (−0.008, 0.007)
Model 3	Reference	−0.002 (−0.006, 0.002)	−0.003 (−0.010, 0.003)	−0.002 (−0.009, 0.006)
Potato fries/chips				
Intake, g/day	2 (1-4)	8 (7-10)	17 (14-21)	42 (32-60)
Model 1a	Reference	0.004 (0.001, 0.007)	0.008 (0.002, 0.014)	0.012 (0.004, 0.020)
Model 1b	Reference	0.003 (0.000, 0.006)	0.007 (0.001, 0.013)	0.010 (0.002, 0.018)
Model 2	Reference	0.003 (0.000, 0.006)	0.007 (0.000, 0.013)	0.010 (0.001, 0.018)
Model 3	Reference	0.002 (−0.001, 0.005)	0.004 (−0.002, 0.011)	0.006 (−0.002, 0.014)

The difference in log means (multiplies of the beta coefficients) and 95% CI are reported for the median intake of each quartile of vegetable intake (g/day) compared with the median intake of first quartile. Model 1a adjusted for age and sex; Model 1b adjusted for age, sex, physical activity level, education level, Socio-Economic Indexes for Areas, income, smoking status, parental history of diabetes, alcohol intake (g/day); Model 2 adjusted for all covariates in Model 1b plus intakes (g/day) of wholegrains, refined grains, red meat, processed meat, poultry, eggs, fish, dairy, fat, fruits, nuts, miscellaneous and discretionary foods; Model 3 adjusted for all covariates in Model 2 plus body mass index and self-reported prevalence of cardiovascular disease.

Vegetable intakes (g/day), postload plasma glucose and fasting blood glucose are given as median (interquartile range).

**Table 3.** Association of vegetables intake with insulin sensitivity and estimates of pancreatic β-cell function (n = 8009)

	Quartiles of vegetable intake			
	Q1	Q2	Q3	Q4
**Serum insulin, µU/mL**				
Total vegetables				
Intake, g/day	75 (57-88)	114 (106-123)	150 (141-161)	208 (189-242)
Model 1a	Reference	−0.010 (−0.027, 0.006)	−0.018 (−0.043, 0.006)	−0.028 (−0.055, 0.000)
Model 1b	Reference	−0.003 (−0.019, 0.013)	−0.007 (−0.031, 0.017)	−0.016 (−0.042, 0.010)
Model 2	Reference	−0.003 (−0.020, 0.013)	−0.007 (−0.031, 0.017)	−0.013 (−0.041, 0.015)
Model 3	Reference	−0.011 (−0.024, 0.002)	−0.019 (−0.037, 0.000)	−0.025 (−0.047, −0.003)
Cruciferous vegetables				
Intake, g/day	6 (3-8)	16 (13-18)	26 (23-30)	47 (39-58)
Model 1a	Reference	−0.015 (−0.032, 0.002)	−0.023 (−0.051, 0.005)	−0.012 (−0.042, 0.019)
Model 1b	Reference	−0.012 (−0.028, 0.004)	−0.019 (−0.046, 0.008)	−0.013 (−0.042, 0.016)
Model 2	Reference	−0.010 (−0.026, 0.006)	−0.016 (−0.043, 0.011)	−0.011 (−0.040, 0.019)
Model 3	Reference	−0.011 (−0.021, −0.001)	−0.015 (−0.036, 0.006)	−0.013 (−0.036, 0.010)
Green leafy vegetables				
Intake, g/day	4 (2-6)	10 (8-11)	15 (14-17)	26 (22-32)
Model 1a	Reference	−0.029 (−0.047, −0.011)	−0.049 (−0.077, −0.022)	−0.068 (−0.098, −0.038)
Model 1b	Reference	−0.018 (−0.035, 0.000)	−0.031 (−0.058, −0.004)	−0.047 (−0.077, −0.018)
Model 2	Reference	−0.012 (−0.030, 0.005)	−0.022 (−0.049, 0.005)	−0.033 (−0.063, −0.003)
Model 3	Reference	−0.010 (−0.024, 0.003)	−0.018 (−0.039, 0.003)	−0.028 (−0.051, −0.004)
Legumes				
Intake, g/day	10 (6-13)	21 (18-23)	32 (29-36)	52 (45-65)
Model 1a	Reference	0.005 (−0.012, 0.022)	0.010 (−0.017, 0.037)	0.017 (−0.013, 0.046)
Model 1b	Reference	0.007 (−0.009, 0.024)	0.013 (−0.013, 0.040)	0.019 (−0.010, 0.047)
Model 2	Reference	0.007 (−0.009, 0.023)	0.013 (−0.014, 0.039)	0.018 (−0.011, 0.047)
Model 3	Reference	0.003 (−0.009, 0.016)	0.005 (−0.015, 0.026)	0.004 (−0.018, 0.027)
Yellow/orange/red vegetables				
Intake, g/day	17 (11-22)	32 (29-35)	46 (42-50)	70 (62-83)
Model 1a	Reference	−0.025 (−0.043, −0.008)	−0.039 (−0.066, −0.013)	−0.039 (−0.068, −0.011)
Model 1b	Reference	−0.017 (−0.034, 0.000)	−0.028 (−0.053, −0.002)	−0.031 (−0.059, −0.003)
Model 2	Reference	−0.017 (−0.034, 0.000)	−0.028 (−0.054, −0.002)	−0.033 (−0.062, −0.004)
Model 3	Reference	−0.020 (−0.033, −0.006)	−0.030 (−0.051, −0.010)	−0.031 (−0.053, −0.008)
Allium vegetables				
Intake, g/day	1 (0-2)	4 (3-4)	7 (6-8)	13 (11-17)
Model 1a	Reference	−0.011 (−0.027, 0.005)	−0.020 (−0.048, 0.008)	−0.023 (−0.053, 0.008)
Model 1b	Reference	0.001 (−0.014, 0.016)	0.002 (−0.025, 0.030)	0.005 (−0.025, 0.035)
Model 2	Reference	0.001 (−0.015, 0.016)	0.001 (−0.026, 0.028)	0.003 (−0.028, 0.033)
Model 3	Reference	−0.005 (−0.017, 0.007)	−0.009 (−0.030, 0.012)	−0.008 (−0.032, 0.016)
Other vegetables				
Intake, g/day	5 (3-7)	13 (11-15)	22 (19-25)	40 (33-51)
Model 1a	Reference	−0.019 (−0.036, −0.003)	−0.036 (−0.063, −0.009)	−0.054 (−0.085, −0.024)
Model 1b	Reference	−0.006 (−0.022, 0.010)	−0.012 (−0.039, 0.015)	−0.024 (−0.054, 0.006)
Model 2	Reference	−0.003 (−0.020, 0.013)	−0.007 (−0.034, 0.020)	−0.013 (−0.045, 0.018)
Model 3	Reference	−0.011 (−0.024, 0.001)	−0.021 (−0.043, 0.000)	−0.033 (−0.058, −0.009)
Potatoes				
Intake, g/day	5 (1-9)	22 (18-27)	43 (37-50)	81 (67-104)
Model 1a	Reference	−0.004 (−0.021, 0.013)	−0.006 (−0.035, 0.023)	−0.002 (−0.033, 0.030)
Model 1b	Reference	0.000 (−0.016, 0.016)	0.000 (−0.028, 0.028)	0.002 (−0.028, 0.032)
Model 2	Reference	0.000 (−0.017, 0.016)	−0.001 (−0.029, 0.027)	−0.003 (−0.034, 0.028)
Model 3	Reference	−0.004 (−0.016, 0.009)	−0.007 (−0.029, 0.015)	−0.011 (−0.035, 0.013)
Potato fries/chips				
Intake, g/day	2 (1-4)	8 (7-10)	17 (14-21)	42 (32-60)
Model 1a	Reference	0.018 (0.006, 0.031)	0.041 (0.014, 0.067)	0.069 (0.034, 0.103)
Model 1b	Reference	0.020 (0.008, 0.032)	0.044 (0.018, 0.070)	0.074 (0.040, 0.108)
Model 2	Reference	0.013 (0.000, 0.025)	0.028 (0.002, 0.055)	0.048 (0.012, 0.084)
Model 3	Reference	0.005 (−0.005, 0.015)	0.012 (−0.009, 0.032)	0.019 (−0.008, 0.047)
**HOMA2-%β**				
Total vegetables				
Intake, g/day	75 (57-88)	114 (106-123)	150 (141-161)	208 (189-242)
Model 1a	Reference	−0.005 (−0.014, 0.005)	−0.009 (−0.023, 0.005)	−0.017 (−0.033, −0.001)
Model 1b	Reference	−0.001 (−0.010, 0.009)	−0.003 (−0.017, 0.011)	−0.010 (−0.026, 0.005)
Model 2	Reference	−0.002 (−0.011, 0.008)	−0.004 (−0.018, 0.010)	−0.009 (−0.026, 0.007)
Model 3	Reference	−0.004 (−0.013, 0.004)	−0.008 (−0.021, 0.005)	−0.013 (−0.028, 0.002)
Cruciferous vegetables				
Intake, g/day	6 (3-8)	16 (13-18)	26 (23-30)	47 (39-58)
Model 1a	Reference	−0.007 (−0.016, 0.003)	−0.012 (−0.028, 0.005)	−0.012 (−0.029, 0.006)
Model 1b	Reference	−0.006 (−0.015, 0.004)	−0.010 (−0.026, 0.006)	−0.012 (−0.029, 0.005)
Model 2	Reference	−0.005 (−0.014, 0.005)	−0.009 (−0.024, 0.007)	−0.010 (−0.028, 0.007)
Model 3	Reference	−0.011 (−0.021, −0.001)	−0.008 (−0.022, 0.007)	−0.011 (−0.026, 0.005)
Green leafy vegetables				
Intake, g/day	4 (2-6)	10 (8-11)	15 (14-17)	26 (22-32)
Model 1a	Reference	−0.015 (−0.025, −0.005)	−0.026 (−0.042, −0.010)	−0.040 (−0.057, −0.022)
Model 1b	Reference	−0.009 (−0.019, 0.001)	−0.016 (−0.032, −0.001)	−0.028 (−0.046, −0.011)
Model 2	Reference	−0.006 (−0.016, 0.005)	−0.011 (−0.027, 0.006)	−0.019 (−0.037, −0.001)
Model 3	Reference	−0.004 (−0.014, 0.005)	−0.008 (−0.023, 0.007)	−0.015 (−0.032, 0.001)
Legumes				
Intake, g/day	10 (6-13)	21 (18-23)	32 (29-36)	52 (45-65)
Model 1a	Reference	0.001 (−0.009, 0.010)	0.001 (−0.015, 0.017)	0.001 (−0.016, 0.018)
Model 1b	Reference	0.002 (−0.008, 0.011)	0.003 (−0.013, 0.018)	0.003 (−0.014, 0.020)
Model 2	Reference	0.001 (−0.009, 0.011)	0.002 (−0.014, 0.017)	0.002 (−0.015, 0.019)
Model 3	Reference	−0.001 (−0.009, 0.008)	−0.002 (−0.016, 0.013)	−0.003 (−0.019, 0.012)
Yellow/orange/red vegetables				
Intake, g/day	17 (11-22)	32 (29-35)	46 (42-50)	70 (62-83)
Model 1a	Reference	−0.007 (−0.017, 0.004)	−0.010 (−0.026, 0.005)	−0.011 (−0.028, 0.006)
Model 1b	Reference	−0.003 (−0.013, 0.007)	−0.005 (−0.020, 0.010)	−0.007 (−0.023, 0.009)
Model 2	Reference	−0.003 (−0.013, 0.007)	−0.006 (−0.021, 0.010)	−0.009 (−0.026, 0.008)
Model 3	Reference	−0.004 (−0.014, 0.005)	−0.007 (−0.021, 0.007)	−0.008 (−0.024, 0.007)
Allium vegetables				
Intake, g/day	1 (0-2)	4 (3-4)	7 (6-8)	13 (11-17)
Model 1a	Reference	−0.010 (−0.019, −0.001)	−0.018 (−0.034, −0.002)	−0.020 (−0.038, −0.002)
Model 1b	Reference	−0.003 (−0.012, 0.006)	−0.006 (−0.022, 0.010)	−0.005 (−0.022, 0.013)
Model 2	Reference	−0.004 (−0.013, 0.005)	−0.006 (−0.023, 0.010)	−0.006 (−0.024, 0.012)
Model 3	Reference	−0.006 (−0.014, 0.002)	−0.011 (−0.026, 0.004)	−0.011 (−0.027, 0.006)
Other vegetables				
Intake, g/day	5 (3-7)	13 (11-15)	22 (19-25)	40 (33-51)
Model 1a	Reference	−0.009 (−0.018, 0.001)	−0.016 (−0.032, 0.000)	−0.025 (−0.043, −0.007)
Model 1b	Reference	−0.001 (−0.011, 0.008)	−0.003 (−0.019, 0.013)	−0.009 (−0.027, 0.009)
Model 2	Reference	0.000 (−0.009, 0.010)	0.000 (−0.016, 0.016)	−0.002 (−0.020, 0.017)
Model 3	Reference	−0.003 (−0.011, 0.006)	−0.005 (−0.020, 0.009)	−0.009 (−0.026, 0.008)
Potatoes				
Intake, g/day	5 (1-9)	22 (18-27)	43 (37-50)	81 (67-104)
Model 1a	Reference	−0.001 (−0.010, 0.009)	−0.001 (−0.018, 0.015)	−0.003 (−0.021, 0.015)
Model 1b	Reference	0.002 (−0.008, 0.011)	0.002 (−0.014, 0.019)	0.000 (−0.018, 0.018)
Model 2	Reference	0.000 (−0.010, 0.010)	−0.001 (−0.018, 0.015)	−0.007 (−0.025, 0.012)
Model 3	Reference	−0.002 (−0.011, 0.007)	−0.004 (−0.020, 0.011)	−0.010 (−0.027, 0.006)
Potato fries/chips				
Intake, g/day	2 (1-4)	8 (7-10)	17 (14-21)	42 (32-60)
Model 1a	Reference	0.007 (−0.001, 0.014)	0.015 (0.000, 0.030)	0.027 (0.007, 0.047)
Model 1b	Reference	0.008 (0.001, 0.015)	0.018 (0.003, 0.033)	0.032 (0.013, 0.052)
Model 2	Reference	0.004 (−0.003, 0.011)	0.009 (−0.007, 0.025)	0.018 (−0.003, 0.039)
Model 3	Reference	0.000 (−0.006, 0.007)	0.001 (−0.013, 0.016)	0.005 (−0.014, 0.025)
**HOMA2-%S**				
Total vegetables				
Intake, g/day	75 (57-88)	114 (106-123)	150 (141-161)	208 (189-242)
Model 1a	Reference	0.009 (−0.008, 0.026)	0.019 (−0.006, 0.043)	0.036 (0.009, 0.063)
Model 1b	Reference	0.006 (−0.011, 0.022)	0.013 (−0.011, 0.037)	0.029 (0.002, 0.056)
Model 2	Reference	0.007 (−0.010, 0.024)	0.014 (−0.011, 0.039)	0.029 (0.000, 0.059)
Model 3	Reference	0.011 (−0.004, 0.027)	0.020 (−0.003, 0.043)	0.030 (0.003, 0.056)
Cruciferous vegetables				
Intake, g/day	6 (3-8)	16 (13-18)	26 (23-30)	47 (39-58)
Model 1a	Reference	0.006 (−0.010, 0.023)	0.011 (−0.017, 0.039)	0.011 (−0.019, 0.042)
Model 1b	Reference	0.003 (−0.013, 0.020)	0.006 (−0.022, 0.034)	0.009 (−0.021, 0.039)
Model 2	Reference	0.003 (−0.014, 0.019)	0.005 (−0.023, 0.033)	0.007 (−0.024, 0.038)
Model 3	Reference	−0.011 (−0.021, −0.001)	0.013 (−0.013, 0.038)	0.013 (−0.015, 0.040)
Green leafy vegetables				
Intake, g/day	4 (2-6)	10 (8-11)	15 (14-17)	26 (22-32)
Model 1a	Reference	0.022 (0.004, 0.040)	0.037 (0.010, 0.065)	0.052 (0.022, 0.082)
Model 1b	Reference	0.015 (−0.003, 0.033)	0.026 (−0.001, 0.054)	0.038 (0.008, 0.068)
Model 2	Reference	0.012 (−0.006, 0.030)	0.02 (−0.008, 0.048)	0.027 (−0.005, 0.058)
Model 3	Reference	0.007 (−0.009, 0.024)	0.013 (−0.013, 0.038)	0.020 (−0.008, 0.049)
Legumes				
Intake, g/day	10 (6-13)	21 (18-23)	32 (29-36)	52 (45-65)
Model 1a	Reference	−0.005 (−0.022, 0.012)	−0.008 (−0.035, 0.020)	−0.006 (−0.036, 0.024)
Model 1b	Reference	−0.005 (−0.022, 0.012)	−0.008 (−0.035, 0.019)	−0.005 (−0.035, 0.024)
Model 2	Reference	−0.005 (−0.022, 0.012)	−0.008 (−0.036, 0.019)	−0.005 (−0.035, 0.025)
Model 3	Reference	−0.003 (−0.018, 0.012)	−0.005 (−0.030, 0.020)	−0.003 (−0.030, 0.024)
Yellow/orange/red vegetables				
Intake, g/day	17 (11-22)	32 (29-35)	46 (42-50)	70 (62-83)
Model 1a	Reference	0.023 (0.005, 0.040)	0.035 (0.009, 0.062)	0.036 (0.007, 0.065)
Model 1b	Reference	0.019 (0.001, 0.036)	0.030 (0.003, 0.056)	0.033 (0.004, 0.061)
Model 2	Reference	0.020 (0.002, 0.037)	0.031 (0.004, 0.058)	0.035 (0.005, 0.065)
Model 3	Reference	0.016 (0.000, 0.032)	0.026 (0.001, 0.050)	0.027 (0.000, 0.055)
Allium vegetables				
Intake, g/day	1 (0-2)	4 (3-4)	7 (6-8)	13 (11-17)
Model 1a	Reference	0.018 (0.002, 0.033)	0.032 (0.004, 0.060)	0.032 (0.001, 0.063)
Model 1b	Reference	0.010 (−0.006, 0.025)	0.017 (−0.011, 0.045)	0.015 (−0.016, 0.046)
Model 2	Reference	0.011 (−0.005, 0.027)	0.019 (−0.009, 0.047)	0.018 (−0.014, 0.050)
Model 3	Reference	0.010 (−0.005, 0.024)	0.018 (−0.008, 0.043)	0.019 (−0.010, 0.048)
Other vegetables				
Intake, g/day	5 (3-7)	13 (11-15)	22 (19-25)	40 (33-51)
Model 1a	Reference	0.011 (−0.005, 0.027)	0.024 (−0.003, 0.051)	0.054 (0.023, 0.085)
Model 1b	Reference	0.002 (−0.015, 0.018)	0.008 (−0.020, 0.036)	0.034 (0.003, 0.065)
Model 2	Reference	0.001 (−0.015, 0.018)	0.007 (−0.021, 0.035)	0.030 (−0.002, 0.063)
Model 3	Reference	0.013 (−0.003, 0.028)	0.025 (−0.001, 0.051)	0.045 (0.015, 0.074)
Potatoes				
Intake, g/day	5 (1-9)	22 (18-27)	43 (37-50)	81 (67-104)
Model 1a	Reference	0.006 (−0.011, 0.023)	0.009 (−0.020, 0.038)	0.005 (−0.026, 0.036)
Model 1b	Reference	0.004 (−0.013, 0.021)	0.006 (−0.023, 0.035)	0.004 (−0.027, 0.035)
Model 2	Reference	0.006 (−0.011, 0.023)	0.010 (−0.019, 0.039)	0.011 (−0.021, 0.043)
Model 3	Reference	0.008 (−0.008, 0.023)	0.014 (−0.013, 0.040)	0.015 (−0.014, 0.045)
Potato fries/chips				
Intake, g/day	2 (1-4)	8 (7-10)	17 (14-21)	42 (32-60)
Model 1a	Reference	−0.019 (−0.032, −0.007)	−0.042 (−0.068, −0.015)	−0.057 (−0.092, −0.022)
Model 1b	Reference	−0.021 (−0.034, −0.008)	−0.045 (−0.071, −0.018)	−0.062 (−0.097, −0.027)
Model 2	Reference	−0.015 (−0.028, −0.002)	−0.032 (−0.060, −0.004)	−0.042 (−0.079, −0.004)
Model 3	Reference	−0.007 (−0.018, 0.005)	−0.014 (−0.039, 0.011)	−0.017 (−0.051, 0.017)

The difference in log means (multiplies of the beta coefficients) and 95% CI are reported for the median intake of each quartile of vegetable intake (g/day) compared with the median intake of first quartile. Model 1a adjusted for age and sex; Model 1b adjusted for age, sex, physical activity level, education level, Socio-Economic Indexes for Areas, income, smoking status, parental history of diabetes, alcohol intake (g/day); Model 2 adjusted for all covariates in Model 1b plus intakes (g/day) of wholegrains, refined grains, red meat, processed meat, poultry, eggs, fish, dairy, fat, fruits, nuts, miscellaneous and discretionary foods; Model 3 adjusted for all covariates in Model 2 plus body mass index and self-reported prevalence of cardiovascular disease.

Vegetable intakes (g/day), serum insulin, HOMA2-%β and HOMA2-%S are given as median (IQR).

For vegetable subgroups, green leafy vegetable intakes were significantly inversely associated with PLG (*P* = .002; *P*_nonlinearity_ = .041; Fig. S3), HOMA2-%β (*P* = .004; *P*_nonlinearity_ = .538; Fig. S3), and serum insulin (*P* = .014; *P*_nonlinearity_ = .497; Fig. S3 (21)). Participants in the highest, compared with the lowest quartile of green leafy vegetable intake had a 4% (ratio of means 0.96 [0.95, 0.98]) lower PLG, 3% (0.97 [0.96, 0.99]) lower HOMA2-%β, and a 5% (0.95 [0.93, 0.98]) lower serum insulin upon multivariable adjustments (Model 1b; Table 2 and Table 3). The associations remained inverse upon further adjustment for all the dietary confounders using all-component model (Model 2; Table 2 and Table 3). The association between cruciferous vegetable intake and PLG assumed a J-shaped pattern wherein participants with moderate intakes of cruciferous vegetable (Q3) had a 2% (0.98 [0.96, 1.00]) lower PLG than those with lowest intakes (Q1) upon multivariable adjustments (Model 1b; Table 2). Higher intakes of legumes and allium vegetables were not associated with any of the glucose or insulin markers. Furthermore, intakes of yellow/orange/red vegetables and remaining vegetables (“other vegetables”) were each significantly inversely associated with PLG (*P* < .001; *P*_nonlinearity_ = .004 and *P* < .001; *P*_nonlinearity_ = .014 respectively; Fig. S4) (21). Participants in the highest compared with the lowest quartile of yellow/orange/red vegetable and remaining vegetable intake observed a respective 4% (0.96 [0.95, 0.98]) and 4% (0.96 [0.94, 0.98]) lower PLG upon multivariable adjustments (Model 1b; Table 2). These associations persisted upon further adjustment for dietary confounders (Model 2; Table 2). Notably, a higher intake of potatoes, excluding fries/chips, was not significantly associated with any of the diabetes markers. Conversely, for potato fries/chips, significant nonlinear direct associations with FPG (*P* = .032, *P*_nonlinearity_ = .048), HOMA2-%β (*P* < .001, *P*_nonlinearity_ = .021) and serum insulin (*P* < .001, *P*_nonlinearity_ = .012) were observed. The association between potato fries/chips and HOMA2-%S was significant and nonlinear with an inverse relationship between the intakes 0 and 20 g/day (*P* = .001, *P*_nonlinearity_ = .003; Fig. S5 (21)). Participants in the highest compared with the lowest potato fries/chips intake quartile had a 1% (1.01 [1.00, 1.02]) higher FPG, 3% (1.03 [1.01, 1.05]) higher HOMA2-%β, and 8% (1.08 [1.04, 1.11]) higher serum insulin but a 6% (0.94 [0.91, 0.97]) lower HOMA2-%S upon multivariable adjustments (Model 1b; Table 2 and Table 3). The associations between potato fries/chips and FPG, serum insulin, and HOMA2-%S remained upon further adjustments of the dietary confounders (Model 2; Table 2 and Table 3).

### Prospective Association Between Vegetable Intake and T2D at 12 Years

**Figure 2.**
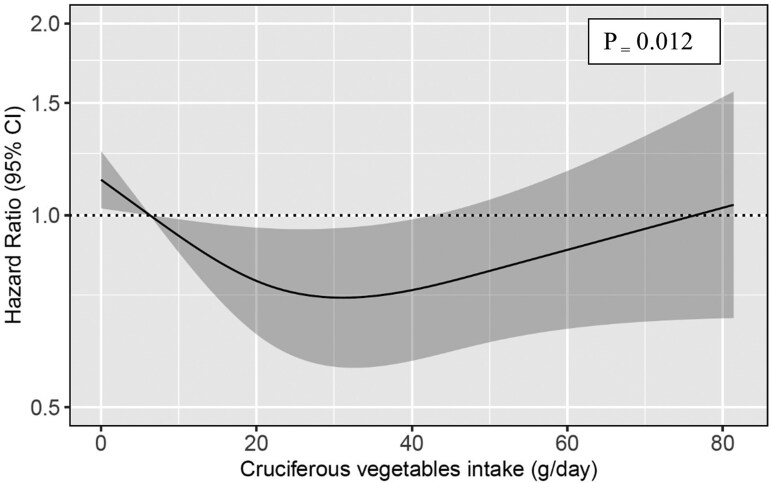
Hazard ratios for the association between cruciferous vegetable intake (g/day) and incident T2D during 12 years of follow-up among the participants of AusDiab study (n = 5107) derived from restricted cubic spline based on Cox proportional hazards model and compare the amount of cruciferous vegetable intake with the median intake in lowest quartile (Q1). Model is adjusted for age, sex, physical activity level, education level, Socio-Economic Indexes for Areas, income, smoking status, parental history of diabetes and alcohol intake (Model 1b). *P* values for the overall effect of cruciferous vegetable intake on the response were obtained using likelihood ratio tests comparing nested models.

Among the 5107 participants with available follow-up data, a total of 342 incident T2D cases were recorded. This included 177 cases at the 5-year follow-up and 165 cases at the 12-year follow-up. At the end of 12-year follow-up, no association was observed between total vegetable intake and incident T2D upon multivariable adjustments (HR_Q4vsQ1_ = 0.97 [0.77, 1.24]; Model 1b, Table S2 (21)). However, within the vegetable subgroups, participants with moderate intakes of cruciferous vegetables (Q3) compared with those with the lowest intakes (Q1) had a 25% (HR_Q3vsQ1_: 0.75 [0.59, 0.95]) lower risk of incident T2D at 12 years upon multivariable adjustments (Model 1b, Table S2 (21)). This association assumed a J-shaped pattern and did not attain significance for the highest quartile, Q4 (*P* = .012; *P*_nonlinearity_ = .006, Fig. 2). Upon further adjustments for dietary covariates and potential mediators, this inverse association strengthened. Participants with the highest cruciferous vegetable intakes (Q4) had a 26% (HR_Q4vs.Q1_: 0.74 [0.57, 0.96]) lower risk of T2D than those with the lowest intakes. No association with incident T2D was observed for the remaining vegetable subgroups, potatoes, or potato fries/chips (Table S2 (21)).

### Effect Modification by Known Risk Factors for T2D

No significant interactions with sex, BMI, or physical activity level were observed for the association between total vegetable intakes and all primary outcomes (*P* > .05). However, a modest interaction was identified between vegetable intake and sex for the association with HOMA2-%S (*P* = .046). Similarly, a negligible interaction was observed between vegetable intake and physical activity level for the association with FPG (*P* = .049).

## Discussion

In this population of 8009 Australian adults from the AusDiab study, a higher total vegetable intake was associated with lower PLG but no other measures of glucose tolerance and insulin sensitivity. Similarly, moderate to high intakes of green leafy, cruciferous, and yellow/orange/red vegetables were associated with lower PLG. Only a higher intake of green leafy vegetables was inversely associated with HOMA2-%β and serum insulin. Conversely, a higher intake of potato fries/chips was positively associated with FPG, HOMA2-%β, and serum insulin and inversely associated with HOMA2-%S. Intakes of legumes, allium vegetables, and potatoes (excluding fries/chips) were not associated with any outcome. Over a 12-year follow-up period, only a moderate intake of cruciferous vegetable was associated with a lower risk of T2D.

The intricate pathogenesis of T2D involves β-cell dysfunction, obesity, and insulin resistance (33, 34). Notably, β-cell dysfunction or insulin resistance may manifest long before elevated blood glucose levels and T2D onset (34, 35). This complex interplay encompasses a decline in β-cell function or their overstimulation, occurring before the onset of obesity and insulin resistance. These processes lead to either hyperinsulinemia or β-cell exhaustion (34, 36), potentially causing impaired FPG and PLG, observed in prediabetes (37), predisposing individuals to an increased risk of T2D in future. In our current study, a higher total vegetable intake was inversely associated with 2-hour PLG, with no association detected with other markers, including FPG, β-cell function (HOMA2-%β), and insulin sensitivity (HOMA2-%S). A potential explanation lies in the chemical composition variations within specific vegetable subgroups (38). It is plausible that the associations might be concealed when aggregated as total vegetables, underscoring the importance of nuanced investigations into vegetable subgroups.

In our study, exploring vegetable subgroups revealed a dose–response inverse association between moderate to higher intake of green leafy, cruciferous, and yellow/orange/red vegetable with PLG. This indicates that participants with higher intake of these specific vegetable subgroups had a lower rise in blood glucose levels after food consumption compared with those with lower intake. In addition, green leafy vegetable intakes were inversely associated with serum insulin and HOMA2-%β, indicating lower insulin secretion activity likely due to higher insulin sensitivity and less β-cell dysfunction among participants with higher intakes (26). Our findings align with a cross-sectional study of 175 overweight Latino youths, which observed 31% higher insulin sensitivity (mean_consumers vs nonconsumers_: 2.1 vs 1.6; *P* = .03) and 25% lower acute insulin response (first rapid insulin secretion in response to glucose; mean_consumers vs nonconsumers_: 1191 vs 1588; *P* = .05) among consumers of nutrient-rich vegetables (dark green and deep yellow/orange vegetables) compared with nonconsumers (39). The exact biological mechanisms underlying the protective effects of vegetable intake against diabetes and its markers remain uncertain, but preclinical studies suggest several pathways. Animal studies indicate that higher vegetable intake helps to reduce body weight, plasma glucose, and insulin resistance, thereby contributing to the regulation of glucose–insulin homeostasis and diabetes prevention (40, 41). Additionally, the synergy of several nutrients and phytochemicals, including dietary fiber found in vegetables, may play a role in preventing diabetes (42). Dietary fiber from vegetables, by delaying gastric emptying (43) and glycemic responsiveness (44), has the potential to reduce inflammation (45) and weight gain (46). Furthermore, green leafy vegetables are rich in many essential nutrients and other bioactive compounds including, but not limited to, β-carotene, lutein, folate, vitamin K_1_, and nitrate (47, 48). Intake of these vegetables, and their beneficial components, has been linked with lower risk of diabetes (9, 42, 49, 50). Sulforaphane, a derivative of glucoraphanin found in cruciferous vegetables, is another component that might help prevent diabetes due to its antioxidant and anti-inflammatory properties (51). A preclinical study has demonstrated that sulforaphane supplementation significantly reduced body weight, FPG, serum insulin, and the HOMA-IR index, improving insulin sensitivity in mice fed a high-fat diet (52). In addition, human studies using concentrated broccoli sprout extract (concentrated sulforaphane) report reduced blood glucose and glycated hemoglobin in obese patients with dysregulated T2D (53). To our knowledge, few studies have explored the association between vegetable intakes and markers of glucose tolerance and insulin sensitivity. While our study showed clinically minor changes in diabetes markers related to glucose tolerance and insulin sensitivity, it sheds light on the physiological alterations in insulin regulation and glucose tolerance resulting from higher vegetable and subgroups of vegetable intake and supports the notion that vegetable subgroups may act differently in regulating insulin and blood glucose levels.

While potatoes have been reported to be positively associated with diabetes (4), this relationship is highly influenced by their preparation and cooking methods as well as the underlying dietary pattern of participants who consume high quantities of potatoes (9). In line with our previous findings that intakes of potatoes (excluding fries/chips) were not associated with incident T2D (9), the current investigation found that a higher intake of potatoes (excluding fries/chips) was not associated with markers of glucose tolerance, β-cell function, and insulin sensitivity. Conversely, a higher intake of potato fries/chips was associated with significantly higher serum insulin and HOMA2-%β but a lower HOMA2-%S, suggesting adverse physiological impacts on glucose tolerance and insulin sensitivity with higher consumption of “unhealthy” potatoes. Studies exploring the association of potatoes with markers of glucose tolerance, β-cell function, and insulin sensitivity are lacking. However, existing evidence, including our own, suggests that higher intake of potato fries/chips is associated with higher risk of T2D (9, 11, 54, 55). Our findings indicate that people with higher consumption of potato fries/chips might experience higher insulin response and insulin resistance, potentially leading to a higher risk of T2D in the future. Studies reveal that potato fries/chips contain higher amount of unhealthy oil, salt, trans fat, and acrylamide, which have been linked with higher body weight, insulin resistance and T2D (56-59). The current findings align with our previous study and emphasize that potatoes, when prepared in a healthy way, may have a neutral impact on markers of glucose tolerance. However, their negative influence on these markers might be amplified if prepared in an unhealthy way, involving the addition of salt and fats.

Our finding suggested an inverse association between moderate cruciferous vegetable intake and T2D, but it did not indicate an association between total vegetable, vegetable subgroup, potatoes, potato fries/chips intake, and T2D. While our cross-sectional findings did not appear to translate to a statistically significant lower risk of T2D after 12 years of follow-up in this cohort—perhaps due to low power and methodological limitations—we do see that intakes of cruciferous, green leafy, and yellow/orange/red vegetables are associated with a lower risk of T2D in other studies (4, 7-9). Biomarkers of vegetable intake have shown an inverse association with T2D risk in a large European study (42). Other studies, including our own, observed a significant lower risk of T2D in participants with the highest intakes of cruciferous and green leafy vegetables (8, 9). The absence of an association between higher cruciferous vegetable intake level and T2D in the present study may be attributed to potential over-reporting of vegetable intakes, particularly among people with unhealthy dietary habits, indicating a potential social-desirability bias. On the contrary, the null association for potatoes aligns with our prior research and another US-based study (9, 60). This finding supports the recent Nordic dietary guidelines recommending the inclusion of potatoes, prepared in a healthy way (“less salt and fat”), into a regular diet (13). However, it is essential to acknowledge that these prospective associations require careful interpretation due to methodological limitations and potential selection bias because of participants lost to follow-up.

The AusDiab study leverages several strengths, including the use of different biochemical markers of glucose tolerance to assess the relationship between vegetable intake and diabetes. The incorporation of several demographic, lifestyle, and dietary confounders to assess the relationship between vegetable intake, diabetes markers, and incidence of T2D adds strength to our study findings. However, the limitations of the study warrant careful consideration. The observational nature of the study precludes the inference of causality. Measurement errors in dietary exposures, coupled with other limitations of the FFQ such as recall bias needs to be considered while interpreting the findings. The over-representation of the study participants from higher education and socioeconomic subgroups (17), along with potential selection bias induced by loss to follow-up in the prospective association limits the generalizability of our findings. Additionally, relying solely on baseline dietary data introduces potential exposure estimation error for the prospective association, as dietary habits may change during follow-up. Lastly, residual confounding cannot be ruled out even though several adjustments were made for lifestyle and dietary covariates. These limitations underscore the need for further investigation using larger and more diverse populations to enhance the generalizability and robustness of our findings.

In summary, our study findings suggest that a diet rich in vegetables, in particular, green leafy, cruciferous, and yellow/orange/red vegetables may help improve glucose tolerance in Australian adults. Moreover, a diet rich in green leafy vegetables might improve insulin sensitivity. Conversely, a higher intake of potato fries/chips, but not potatoes prepared in a healthy way, may worsen glucose tolerance and insulin sensitivity. These findings indicate a nuanced relationship between vegetable subgroups, glucose tolerance and insulin sensitivity.

## Data Availability

Restrictions apply to the availability of some, or all data generated or analyzed during this study to preserve patient confidentiality or because they were used under license. The corresponding author will on request detail the restrictions and any conditions under which access to some data may be provided.

## Abbreviations

BMI: body mass index
FFQ: Food Frequency Questionnaire
FPG: fasting plasma glucose
HOMA2-%β: homeostasis model assessment of β-cell function
HOMA2-%S: HOMA2 of insulin sensitivity
PLG: postload plasma glucose
T2D: type 2 diabetes
